# A quixotic view of spatial bias in modelling the distribution of species and their diversity

**DOI:** 10.1038/s44185-023-00014-6

**Published:** 2023-05-03

**Authors:** Duccio Rocchini, Enrico Tordoni, Elisa Marchetto, Matteo Marcantonio, A. Márcia Barbosa, Manuele Bazzichetto, Carl Beierkuhnlein, Elisa Castelnuovo, Roberto Cazzolla Gatti, Alessandro Chiarucci, Ludovico Chieffallo, Daniele Da Re, Michele Di Musciano, Giles M. Foody, Lukas Gabor, Carol X. Garzon-Lopez, Antoine Guisan, Tarek Hattab, Joaquin Hortal, William E. Kunin, Ferenc Jordán, Jonathan Lenoir, Silvia Mirri, Vítězslav Moudrý, Babak Naimi, Jakub Nowosad, Francesco Maria Sabatini, Andreas H. Schweiger, Petra Šímová, Geiziane Tessarolo, Piero Zannini, Marco Malavasi

**Affiliations:** 1 BIOME Lab, Department of Biological, Geological and Environmental Sciences, Alma Mater Studiorum University of Bologna, via Irnerio 42, 40126 Bologna, Italy; 2 Czech University of Life Sciences Prague, Faculty of Environmental Sciences, Department of Spatial Sciences, Kamýcka 129, Praha - Suchdol, 16500 Czech Republic; 3 Department of Botany, Institute of Ecology and Earth Science, University of Tartu, J. Liivi 2, 50409 Tartu, Estonia; 4 Evolutionary Ecology and Genetics Group, Earth and Life Institute, UCLouvain, 1348 Louvain-la-Neuve, Belgium; 5 CICGE (Centro de Investigação em Ciências Geo-Espaciais), Universidade do Porto, Porto, Portugal; 6 Biogeography, BayCEER, University of Bayreuth, Universitaetsstraße 30, 95440 Bayreuth, Germany; 7 Georges Lemaître Center for Earth and Climate Research, Earth and Life Institute, UCLouvain, Louvain-la-Neuve, Belgium; 8 Department of Life, Health and Environmental Sciences, University of L’Aquila, Piazzale Salvatore Tommasi 1, 67100 L’Aquila, Italy; 9 School of Geography, University of Nottingham, Nottingham, UK; 10 Dept of Ecology and Evolutionary Biology, Yale University, New Haven, CT USA; 11 Center for Biodiversity and Global Change, Yale University, New Haven, CT USA; 12 Knowledge Infrastructures, Campus Fryslan University of Groningen, Leeuwarden, The Netherlands; 13 Department of Ecology and Evolution, University of Lausanne, 1015 Lausanne, Switzerland; 14 Institute of Earth Surface Dynamics, University of Lausanne, 1015 Lausanne, Switzerland; 15 MARBEC, Univ Montpellier, CNRS, Ifremer, IRD, Sète, France; 16 Department of Biogeography and Global Change, Museo Nacional de Ciencias Naturales (MNCN-CSIC), Madrid, Spain; 17 University of Leeds, Leeds, UK; 18 University of Parma, Parma, Italy; 19 UMR CNRS 7058 “Ecologie et Dynamique des Systèmes Anthropisés” (EDYSAN), Université de Picardie Jules Verne, 1 Rue des Louvels, 80000 Amiens, France; 20 Department of Computer Science and Engineering, Alma Mater Studiorum University of Bologna, via Irnerio 42, 40126 Bologna, Italy; 21 Rui Nabeiro Biodiversity Chair, MED Institute, University of Évora, Évora, Portugal; 22 Institute of Geoecology and Geoinformation, Adam Mickiewicz University, Krygowskiego 10, 61-680 Poznan, Poland; 23 Faculty of Forestry and Wood Sciences, Czech University of Life Sciences Prague, Prague - Suchdol, Czech Republic; 24 Department of Plant Ecology, Institute of Landscape and Plant Ecology, University of Hohenheim, Stuttgart, Germany; 25 Federal University of Goiás, Campus Central, Anápolis, Brazil; 26 University of Sassari, Department of Chemistry, Physics, Mathematics and Natural Sciences, Sassari, Italy

**Keywords:** Biodiversity, Biogeography, Community ecology, Ecological modelling

## Abstract

Ecological processes are often spatially and temporally structured, potentially leading to autocorrelation either in environmental variables or species distribution data. Because of that, spatially-biased in-situ samples or predictors might affect the outcomes of ecological models used to infer the geographic distribution of species and diversity. There is a vast heterogeneity of methods and approaches to assess and measure spatial bias; this paper aims at addressing the spatial component of data-driven biases in species distribution modelling, and to propose potential solutions to explicitly test and account for them. Our major goal is not to propose methods to remove spatial bias from the modelling procedure, which would be impossible without proper knowledge of all the processes generating it, but rather to propose alternatives to explore and handle it. In particular, we propose and describe three main strategies that may provide a fair account of spatial bias, namely: (i) how to represent spatial bias; (ii) how to simulate null models based on virtual species for testing biogeographical and species distribution hypotheses; and (iii) how to make use of spatial bias - in particular related to sampling effort - as a leverage instead of a hindrance in species distribution modelling. We link these strategies with good practice in accounting for spatial bias in species distribution modelling.

## Introduction

’A greater acknowledgement of model uncertainty often has the consequence of widening our uncertainty bands [...]. Since hedging against uncertainty is hard work, this is an unpopular turn of events, at least in the short run. But [...] which is worse - widening the bands now, or missing the truth later?’^1^

Ecological processes are often spatially and temporally structured, so both environmental variables and species observations can potentially be autocorrelated^2,3^. Modelling the geographic distribution of species and the composition of ecological communities is key to preserve biodiversity and support a proper management of the habitats in which species live and have adapted over their evolutionary history^4–7^. From this point of view, predicting the distributions of species and communities in space and time provides a powerful tool for conservation planning^8–12^. Hence, studying species distribution changes might represent an effective approach to understand the complex interplay between the current biodiversity crisis and anthropogenic climate change^13–15^.

Yet, complete knowledge of the distribution of any plant or animal species, and how these aggregate into more or less diverse communities, is hardly achievable. In some cases, the battle of ecologists against the many problems related to the modelling of species distributions becomes quixotic, or similar to fighting against a chimera. Hence, it needs to be approached in an idealistic way, fostering new ideas to fight the many challenges associated with biodiversity modelling^16^. Such a battle requires proper modelling approaches, which simultaneously account for empirical evidence^13^ and stochastic processes^15^. In this context, Species Distribution Models (hereafter SDMs, also known as Ecological Niche Models, Habitat Suitability Models and many other names used in the scientific literature^9,17^) are powerful tools, since they provide insights into species or community distributions in space and their potential shifts over time^18^. In practical terms, depending on the final interest or overarching goal, the label might change, e.g., labelling it SDMs when the focus is on the spatial distribution of species and labelling it ENMs when the focus is on the underlying drivers, namely the niche requirements of species^19^.

In this paper we did not explicitly distinguish between SDMs, ENMs or HSMs, the three main acronyms used for the same underlying model machinery, since they are all relying on the estimation of ecological requirements of species for predicting their distributions in space and time^19^. In addition to that, other labels such as Potential Habitat Distribution Models (PHDMs), Climate Envelope Models (CEM), Resource Selection Functions (RSF) and others are also used to name this category of niche- or habitat-suitability based distribution models. In fact, we share the view that niches—or habitats—should not be distinguished from or opposed to distributions because these two are faces of the same coin, where the coin is a species with one side being the distribution within the geographical space and the other side the niche as an envelope of habitat suitability within the environmental space. Hence, in our opinion, niche, habitat suitability and distribution are too much entangled to dissociate them into separate entities or types of models (see ref. ^20^ for an example of such an entanglement focusing only on SDMs and ENMs).

All SDMs typically rely on (i) species distribution data, either in the form of both presence and background data (also called pseudo-absences) or presence and true absence data gathered in the field, as well as (ii) a list of predictor variables expected to represent the ecological and geographical drivers of the species’ distribution range^17^. Understanding the spatial covariation of species and their assembly into communities is crucial in ecology^21,22^, so a wealth of methodological approaches has been developed recently to account for species co-occurrences; for instance, joint SDMs—for modelling the covariance of multiple species together (e.g., ref. ^23^)—or stacked SDMs—for modelling single species distributions sequentially and combining them afterwards (e.g., ref. ^24^)—can be used to estimate community-level parameters like species richness^25,26^. In other words, properly stacking SDMs and considering the biotic interactions among species^24,27^ will yield more realistic estimates of spatial patterns in alpha diversity that relate to environmental gradients^28^. This said, no modelling techniques are free from the uncertainty coming from biases in the input data, like uneven sampling effort^29–35^ or spatial positioning errors^36–38^. Here, an integration of species distributions and community-level biodiversity modelling can be performed under the Spatially-Explicit Species Assemblage Modelling framework (SESAM^39^), in which species associations and biotic interactions are explicitly considered^40^.

The increasing availability of spatially explicit open-access databases on species distribution and community composition (e.g., GBIF, sPlotOpen) with appropriate georeferencing^41–43^—coupled with technical and methodological advances for data querying, cleaning, and analysis—opened up new opportunities for global species distribution modelling^44–46^. Furthermore, large-scale environmental layers describing bioclimatic and edaphic conditions have been effectively used as proxies of ecological and climatic drivers of species distributions^47^. These global gridded data, under a structured framework, have been used to systematically select proper environmental variables from a large suite of spatio-environmental variables^48^. Nonetheless, the actual knowledge on species distributions over wide geographical regions is still far from being complete^49–54^, and suffers from pervasive geographical biases^55–64^.

Projecting species distributions for regions and time periods other than those used during model calibration (i.e., model extrapolation)—based on, e.g., bioclimatic variables—requires explicit recognition of all the possible sources of spatial bias, or the use of mechanistic models of species distribution^65^. In fact, transferring model rules onto non-analogous bioclimatic conditions is perilous and a very risky business^66–68^. In other words, extending such projections to new regions involves some sort of extrapolation risk, simply because the recorded occurrences used for model calibration are incomplete or spatially biased, thus increasing spatial uncertainty^69–71^. For instance, methods would be needed to minimize the effects of spatial autocorrelation among records within the geographical space^72–75^, although in some cases spatial autocorrelation could have minimal effects in peculiar regions, such as in topographically rugged landscapes^76,77^. More generally, starting from spatially biased in-situ samples (or predictors), undesired model outcomes can be expected^78^.

This paper aims at addressing the spatial component of data-driven biases in species distribution modelling, and at proposing potential solutions to explicitly test and account for it. Our major goal is not to propose existing or new methods to remove spatial bias from the modelling procedure, which would be impossible without a proper knowledge of all the processes generating it, but rather to propose alternatives to explore and handle it. In particular, we describe three main strategies that may provide a fair account of spatial bias, namely: (i) how to represent spatial bias; (ii) how to build null models based on virtual species for testing biogeographical and species distribution hypotheses; and (iii) how to make use of spatial bias—in particular related to sampling effort—as a leverage instead of a hindrance in species distribution modelling. In each one of these sections we outline what would be good practices to account for spatial bias in species distribution modelling.

## Visualizing spatial bias in the distribution of species and their diversity

Recently, the massive increase in the availability of biodiversity data^43^, coupled with enhanced computing power and modelling techniques, has fostered a new wave of large-scale analyses of biodiversity patterns^45,79,80^. Nonetheless, data quality plays a crucial role in this process^54,81^. In fact, biodiversity knowledge is often skewed toward specific taxonomic groups^82^, wealthy regions of colonial history^64^, English-speaking research^83,84^, and/or environmental domains^14,82,85,86^, which are the major issues among the so-called seven shortfalls of biodiversity data^59^.

**Fig. 1:**
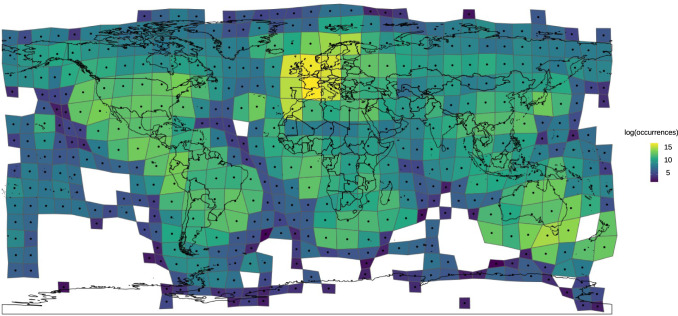
Plant species occurrences over the globe available in GBIF (https://www.gbif.org, latest access: December 2021). The cartogram or density-equalizing map as proposed by Dorling^200^ and Gastner and Newman^201^) shows a bias on species occurrences towards continents with higher sampling effort. To generate the cartogram, a geographical grid of 10 degrees was superimposed on the dataset and the grid cells were further distorted according to the amount of plant species occurrences.

The undersampling of some geographical areas—named the ‘Wallacean shortfall’ by Lomolino^49^ (see also ref. ^87^)—was recently recognized as one of the main factors preventing an exhaustive large-scale understanding of biodiversity patterns^54,88^. Even when biodiversity data are available for a well-studied taxonomic group, these might suffer from a number of bias sources, just to cite a few^33,75,89–91^: lack of standardized sampling design, inconsistent spatial scales, inadequate environmental coverage of the surveys, and observer’s/recorder’s bias (e.g., proximity to roads). Indeed, the large variety of standardized and unstandardized sampling schemes used to survey the distribution of different biological groups often adds up as an additional source of heterogeneity in the data, which may increase the spatial bias and thus affect the complex exercise of modelling species distributions. Likewise, site accessibility and proximity to roads, also have strong effects on data quality, biodiversity inventories being more intensive in locations closer to research centres, infrastructure, highways or places allowing easier access^92–96^. Moreover, the striking geographic bias in the accessibility to resources and in scientific data processing among different regions across the globe can only increase gaps in the data. Altogether, bias in data quality represents a key issue in current macroecological and biogeographical research^97^, and hinders realizing the full potential of using large-scale databases in biodiversity modelling (ref. ^59,96,98,99^, see Fig. 1). Examples exist where smooth geographical biases could be controlled during modelling procedures, in case some in-situ data have still been sampled even in remote areas^100^, but spatial lack of information and stronger bias is generally expected to severely hinder final results^101^.

Spatial bias has been shown to increase uncertainty in the data^91^, and has strong effects on the outcomes of the whole modelling process^78,102,103^. For example, species-people correlations, in which more populated regions show higher biodiversity simply because they are more thoroughly surveyed, are now well known^104^. Ensuring an adequate sampling design is of utmost importance to avoid the generation of truncated species response curves^31,105^. For instance, datasets biased towards widespread environmental conditions across the study area^106–108^ hampered the characterization of species responses to the effects of land transformation or the rarest climate conditions in highly dynamic landscapes such as the Brazilian Atlantic Forest^109^. Moreover, the autecology of species and related eco-geographic characteristics such as species traits^110^, range size^111^ and species niche breadths (i.e., generalist vs specialist^112^), among other factors, can ultimately influence the performance of species-related models^35^.

Recent methodological advances have been proposed to limit spatial bias in data distribution. These can apply either when sampling new species data, or by resampling available data inside a strongly biased dataset^30,43,113^, and restricting analyses to the geographical regions holding enough data coverage^114^. For instance, Hattab et al.^115^ developed a scheme that, by ensuring a systematic sampling of field observations within the environmental conditions available across the study area, can aid in limiting potential shortcomings when modelling species distribution while being not in equilibrium with the contemporary environment (e.g., the case of a recent introduction of an invasive alien species). Likewise, Lembrechts et al.^116^ developed a new framework to design standardized microclimate networks able to capture the largest variation in microclimate at regional or national extents^117,118^.

To appropriately map large-scale patterns of species distributions, the spatial structure of sampling bias must be first understood^63^. For instance, direct gradient analysis^119^ might be used to relate the sampling effort of a focal species distribution with the assumed continuous variation of spatial predictors^120^. In some cases, spatial bias can be attenuated by (i) reducing the clustering of presences within the geographical space^108^ using approaches such as spatial data thinning^121^ or background thickening^34^, or (ii) tuning the model before predicting species distributions^122^. For instance, even in the case of data which are geographically biased, regularization of the models can lead to high quality outputs. As an example, when clumping depends on sampling bias, using spatial or environmental filtering^123^ or rarefaction methods before running SDMs may amend the final output^124^. Concerning spatial data thinning^33^, it might decrease the probability of retaining species with unique environmental conditions. However, in case of a gradual species response to environmental gradients, there is a high model sensitivity to an inappropriate use of data thinning in the environmental space, based on e.g., thresholding methods^125^. From this point of view, a blind data thinning without testing model sensitivity is strongly discouraged. Hence, for instance, proper model averaging might reduce prediction errors^126^. Besides, the combination of predictions derived from different algorithms has generated much attention under the ensemble models umbrella^127^, although in some cases ensemble models might not outperform well-tuned individual models based on machine learning algorithms such as Random Forests or Boosted Regression Trees^128^.

Another important effect of sampling bias is that it creates information gaps^129,130^. This could be solved with recourse to citizen science, although it is well known that such information is even more biased (i) spatially, e.g., with a higher amount of data near roads, cities, research centres, in peculiar ecosystems or regions and, more globally, in the northern hemisphere, but also (ii) taxonomically, toward certain charismatic groups, e.g., vertebrates in terrestrial ecosystems^94,96,131^. In order to solve sampling completeness issues, new tools are now available based on diversity estimates and further fine-tuning of datasets, before they are used for further analysis. As an example, Lobo et al.^132^ propose a tool to estimate the degree of completeness in biodiversity surveys in each territorial unit, when the number of records (including repeated species) is available, as a surrogate of sampling effort. After having estimated the relationship between the number of records and cumulative species richness, Lobo et al.^132^ suggest that the slope of the species accumulation curves and completeness percentages can be used to distinguish and map the level of survey per territorial unit. A similar approach has been proposed by Mokany et al.^133^ based on alpha- and beta-diversity models to measure data completeness. When the number of records is not available for each territorial unit, another approach consists in dividing the study area into regions with known differences in the levels of survey effort. Models can then be computed on these different regions, to check if the observed relationships are consistent among them^104^, obviously provided that all the considered regions span the entire species niche to avoid niche truncation^105,134^.

Finally, it is also of primary importance to reveal the uncertainty in distributional data underlying SDMs, which can be achieved by maps of ignorance accounting for different sources of errors, such as data quality, time elapsed among the field observations, inventory completeness and the eco-geographic distance between species presences and absences (including true absences or pseudo-absences)^53,75,101,135,136^. More recently, Konig et al.^137^ suggested a framework to increase the integration of biodiversity data across domains and resolutions (e.g., from point occurrences to entire floras) for scalable and integrative biodiversity research, especially when the quality of primary data can be integrated with expert knowledge^138^.

## Using virtual species to highlight potential spatial biases of SDMs

In most cases, there is no complete information about the ‘reality’ of the focal species distribution besides the data collected in-situ^101^. This is partly because the completeness of the data extracted from surveys (recorded in-situ) is difficult to measure^139^.

For instance, occurrence data from natural history collections, such as museum or herbaria collections, tend to be very incomplete with a relatively high amount of false absences—i.e., species occurrences missed by the observer in the field in case of a rare or difficult to identify species (see ref. ^140^ on detection bias). Such incompleteness affects our ability of detecting the real spatial coverage of the samples and records available for modelling^141^. These limitations, in turn, can seriously flaw final results of species distribution models, by distorting the relationship between species occurrences and the underlying environmental patterns^56,142^. Yet, quantifying sources of error is essential for proper descriptive or mechanistic modelling of species distributions^143^.

Making use of simulated or in-silico datasets—the so-called ‘virtual ecologist’ approach^143^—allows to generate distribution data with known ecological characteristics^76^, considering that virtual species are better at rejecting candidate models than they are at supporting them^143–147^. The use of virtual species is burgeoning in ecology to build toolkits implementing in-silico analytical experiments simulating natural processes, thanks to the complete control on the configurations of factors constraining the distributions of species^19^. Moreover, virtual species allow creating simulated data for benchmarking models of different complexity. This is true passing from traditional SDMs projecting simple distributions, to those including population dynamics (the so-called hybrid models^148^, see also ref. ^149^ on population dynamics and regulation), up to hierarchical Bayesian process-based dynamic range models^150^, considering that model complexity can impact the projection of species distributions^151^.

Making use of virtual species data allows (i) controlling for random variation in species distributions as well as (ii) simulating patterns of distribution based on known relationships with, for instance, climatic variables (i.e., by species response curves). Due to the artificial nature of such data, the expected underlying processes shaping species distribution patterns can be adjusted or, at least, balanced to account for random or systematic noise^152^. The use of such spatially explicit simulated data helps reaching a better conceptualization and implementation of modelling techniques, leading to the creation of a dominant paradigm for robust generalization and further recommendations for conservation planning. This is difficult with empirical studies, mainly due to confounding effects of interactions among different data types, environmental variables, and methodologies to assess model accuracy^145,153^. Further, models simulating virtual scenarios based on different ecological processes can be used to assess the sensitivity of different SDM algorithms to the effects of historical processes on species distributions^154^.

**Fig. 2:**
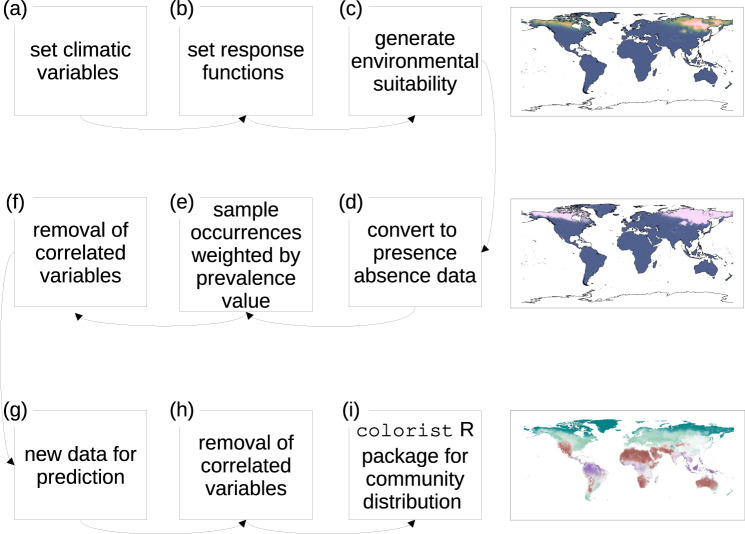
The procedure used to generate virtual species and colorist-based community distribution. First of all, the climatic variables are selected (**a**) and the species response functions of each environmental variable are set (**b**). The environmental suitability of the virtual species distribution is generated in conformity with the response functions (**c**). Then, a logistic conversion transforms it into presences and absences (**d**) and presence and absence points are sampled according to the sample prevalence value (**e**). Furthermore, a collinearity test is performed and the correlated variables are removed (**f**). Once the statistical model has been calibrated, the climatic variables for the prediction are selected (**g**) and—among them—those which are correlated are deleted (**h**). Eventually, multiple virtual species distributions are combined together in colorist R package to map community distribution (**i**). Results are shown in Fig. 3. The complete code to generate virtual species and final maps is available in both Appendix 1 and at the following GitHub repository link: https://github.com/ducciorocchini/Virtual_species_SDM/.

**Fig. 3:**
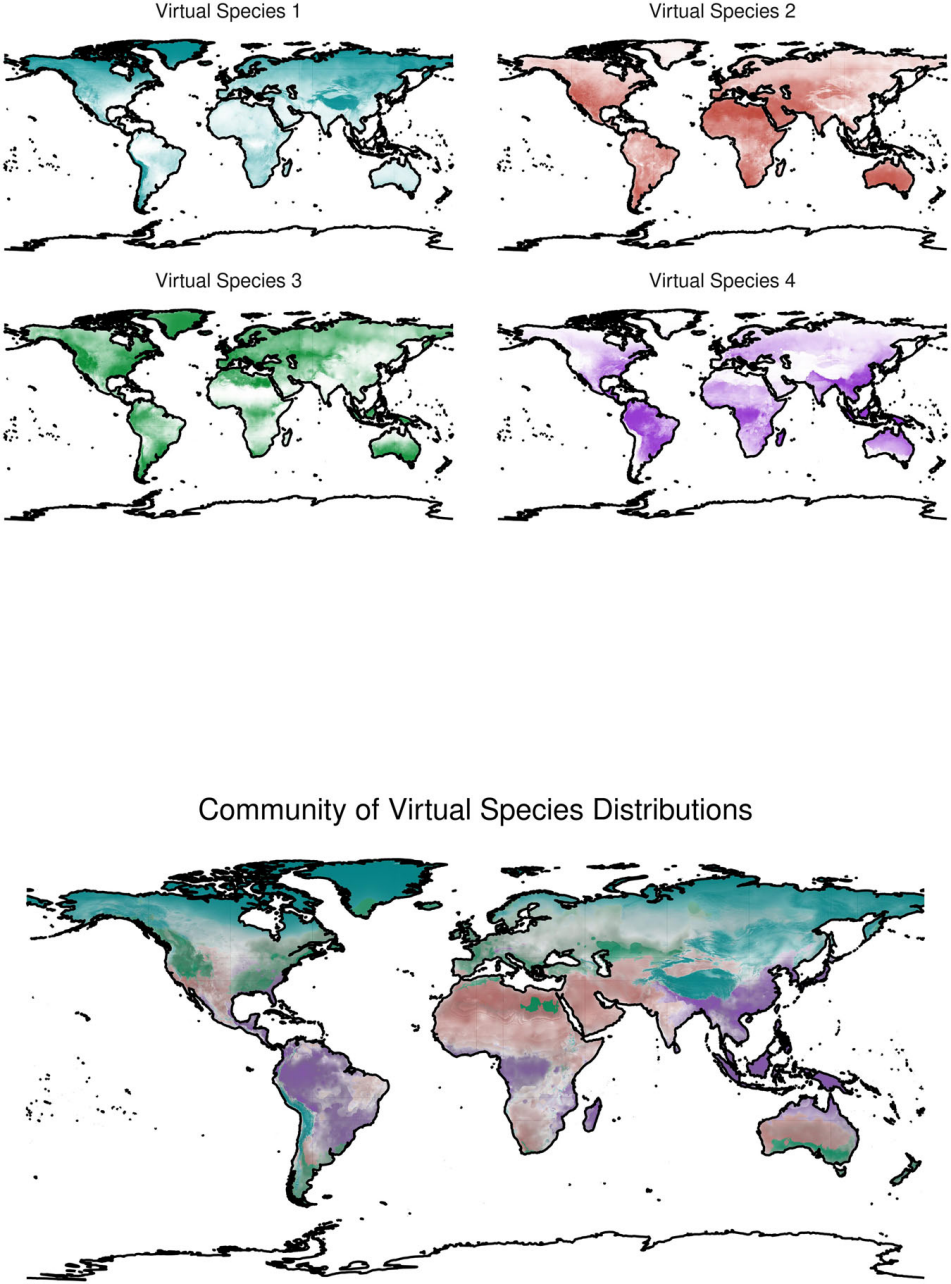
Virtual species can be built to form a virtual community. Starting from colours of single virtual species distributions and relying on the colorist package, it is possible to spatially merge colours and their overlaps in a final gamut which account for single species colour intensity.

From this point of view, open-source spatial algorithms have been developed and are freely downloadable (e.g., refs. ^152,155,156^). We also provide an example in R in Figs. 2 and 3, with the complete code in Appendix 1 or in the following GitHub repository: https://github.com/ducciorocchini/Virtual_species_SDM/(see also ref. ^20^ for a similar example). The concept of virtual species is not the only example of virtual individuals/surfaces, since it has been widely used in disciplines other than ecology—e.g., in geology, virtual globes have been used for geophysical modelling^157^.

Passing from species to assemblages, virtual communities can be simulated (Figs. 2 and 3) to understand what should be an effective sampling effort to predict the distribution of species assemblage, for instance when stacking separate species distribution models^146^. This is generally done by simulating virtual species in a community given a certain virtual species richness, and then manipulating this artificial set by changing different sampling parameters such as sample size, sampling strategy or different species distribution modelling algorithms such as Generalized Linear Models, Generalized Additive Models, MaxEnt, Boosted Regression Trees or Random Forests^146^. This approach is particularly useful, since it allows to better understand species co-existence, which is a long-lasting theme^158^ and (still) an open question^159,160^ in ecology. Furthermore, simulations of different sampling design strategies by virtual communities represent a solid basis for developing experimental designs, which guarantee a high reproducibility and avoid low statistical power due to e.g., small sample size^152^.

Operationally speaking, hitherto there is no consensus about the best methods for generating virtual species distributions. Various examples exist based on: (i) model-based simulations; (ii) model fitting to in-situ data; or (iii) predefined theoretical response (see ref. ^76^). In some cases, it is possible to combine several virtual species to compose a community^146^. Starting from a set of environmental combinations, e.g., using a Principal Components Analysis (PCA) to reduce the number of dimensions of the environmental space, the overlap among niches of different virtual species can be set and controlled to look at potential complements with a focal species of interest^161^. This procedure allows understanding patterns at the community level and balancing potential spatial sampling bias related to rare species. A complete review on the backbone of the virtual species approach is provided by Miller^144^ and Meynard et al.^147^. An experimental approach to data science requires that simulations are a key elements of experimental tests^162–164^. In this paper, we provided an operational way of generating virtual species; albeit we rely on a synthetic and simplistic community of four virtual species, more complex communities composed by thousands of virtual species can be created^165–168^. Further, there is already a broad spectrum of methods for implementing virtual species^147^.

## Sampling effort bias as a covariate in SDMs

Uneven sampling effort is a crucial source of spatial bias. For instance, many areas over the planet are oversampled due to their higher accessibility and closeness to research institutes and universities. On the other hand, most remote areas are undersampled, mainly due to inaccessibility and/or inhospitality to humans^92,169^. The effect of this spatial non-stationarity (see refs. ^170–172^) is a spatial bias in the perceived species distribution and diversity patterns over the planet^173–175^, and therefore a limited coverage of niche-based responses to the environment for many species^56^. If undersampled areas are included in the modelled region, such spatial bias can lead to zero inflation—related to true or false absences in the data—which is problematic to handle^176^. Flexible methods are therefore required to face data with proportions of zeros larger than those expected from pure count Poisson data^177^. This said, zero inflation is not necessarily due to a bias in the species data, but it is often simply an inherent property of ecological systems, where a large number of species are infrequent or rare. Individuals belonging to rare and/or elusive species might be missed, also depending on the strategy of the sampling design adopted. In other words, species distribution models are expected to show a diverse sensitivity to sampling effort, depending on the taxonomic group whose distribution they attempt to forecast^178^.

Unbiased estimates of species distributions are strictly related to the assumption of a random distribution of sampling effort over the area under study. This is also true considering that, when using SDMs to make inference, any model is wrong in its intrinsic definition^176^, but some are less wrong than others and can still provide useful outputs. Sampling effort is also inherently related to scale: species occurrence and community diversity are generally scale dependent. Various approaches have been used to investigate the scale-dependency of ecological variables, from nested sampling^179^ to distance-based sampling^176^. However, these do not guarantee that sampling effort is explicitly measured and/or controlled for. This is particularly true when considering the covariance of different variables^180^—in our case, as an example, of different species. Using mixed-effects or hierarchical models in SDMs, e.g. grounded in the spatial Mixed-effects Models (spaMM) framework^181,182^, should help solving such bias by accounting for pseudo-replication issues.

Obviously, additional causes of uncertainty might increase the spatial bias of species distribution models. For instance, taxonomic misidentification and phenological mismatches of species can lead to highly unreliable models if the biological subject of analysis and the sampling period are not adequately defined^183^, e.g., by sampling a site at the wrong time period or by using an outdated taxonomy^184^. Yet, while these and other sources of uncertainty have non-negligible effects on SDMs accuracy, their impact is normally smaller compared to that of sampling effort^185^, as it may mainly affect the interpretation of the resulting models^183^.

Accounting for uncertainty in SDMs may increase their reliability and predictive power^186^. Based on the above, making use of sampling effort estimates as covariates directly into SDMs can certainly increase their accuracy^174,187–189^. These estimates of sampling effort can be based on (i) the accessibility of the surveyed areas; (ii) time spent on single plots; (iii) multiple visiting periods to catch the right phenological period; (iv) the number of records (including repeated species) per territorial unit; or (v) the number of occurrences within the same taxonomic group, e.g. the genus or family that the focal species belongs to. Such estimates of sampling effort can then be included as covariates in the analysis^190–192^. Similarly, estimates of completeness (e.g., ref. ^132^) or multivariate estimates of data-driven uncertainty, such as the previously cited maps of ignorance approach^75,101^, can be used as ancillary predictors in SDMs, or as spatially-explicit error terms in regression-based modelling techniques^186^.

**Fig. 4:**
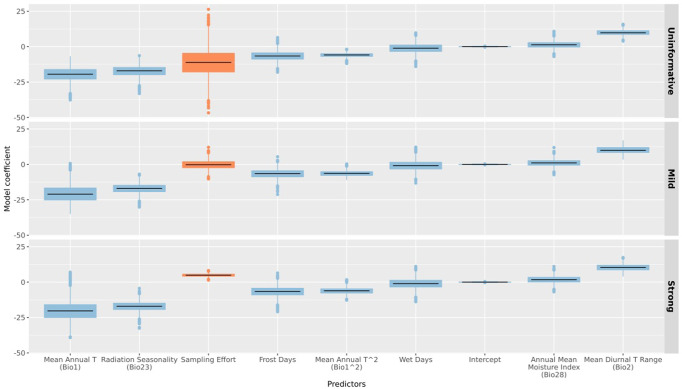
Boxplots of the *β* coefficients in three different models using a different prior on sampling effort. Each box represents the 1st and 3rd quartiles of a coefficient distribution, the black horizontal line the distribution median, the whiskers the limits of the 1.5*interquartile range, while the filled circles represent the outlying points. We showed in red the boxplots reporting the distribution of the *β* coefficient of the sampling effort. Relying on Bayesian statistics it is possible to set three priors on sampling effort: not considering its effect, considering its effect in a mild manner, or in a strong manner. Sampling effort can be measured as an example by the number of revisiting dates. The precision of sampling effort increased passing from the model with an uninformative prior on sampling effort, through that with a mild prior, reaching its highest value in the model with a strong prior. Controlling sampling effort bias using a strongest prior could lead to the comparability of models related to species with opposite degrees of sampling effort in an area. See the main text for additional information. From Rocchini et al.^175^: License Number: 5495740269939, License date: Feb 25th 2023, Licensed Content Publisher: Elsevier.

Starting from the intuitive assumption that a higher sampling effort could be related to intrinsically higher prevalence of species’ occurrence data inside a region, Bayesian inference can integrate this information in the modelling of species distributions to guide model predictions. However, Bayesian methods are, in general, computationally intensive, which makes them sometimes unfeasible for many species over large areas. Alternatively, one can generate very simple covariates to capture the effect of sampling effort in traditional SDMs. For instance, Wasof et al.^193^ fitted SDMs for vascular plant species that included several covariates: a region effect (Alps vs. Fennoscandia) to test potential differences in distribution patterns between the two investigated regions and a covariate reflecting sampling effort based on the total number of presence/absence records available per sampled grid cell (1 km^2^) to account for the spatially imbalanced data within each of the two investigated regions. Furthermore, Rocchini et al.^175^ included sampling effort as a hyper-prior in a multilevel model structure, by considering different degrees of association between sampling effort at large spatial extents to predict the probability of species presence (*Abies alba* over Europe) in smaller nested areas. Sampling effort was estimated as the number of revisiting dates and used for further modelling in three main manners: no effect, mild effect and strong effect. The model with the strongest importance assigned to sampling effort significantly corrected final results for sampling effort bias (Fig. 4). This indicates that sampling effort might be used to supplement the often incomplete information provided by species presence at fine spatial scales. This modelling approach could also be extended considering similar species characterized by opposite degrees of sampling effort in an area (or even the overall species sampling effort; see ref. ^194^). Data on sampling effort for a well surveyed and widespread species could also be considered to correct model outputs for a similar, but less sampled, species belonging to, e.g., the same genus^102^.

## Conclusion

In this short essay, we have addressed a range of methods to quantify and account for spatial bias when mapping species distribution and diversity (see also ref. ^195^). Based on this general overview of the issues related to spatial bias in modelling species distribution, we basically propose (i) to integrate several methods to set the best tuning and achieve optimal model complexity when modelling distributions of species and their relative diversity^196,197^ as well as (ii) to find the most effective visualization techniques to explore model behaviour^198^.

If left unchecked, spatial bias could impair species distribution models/outputs, thereby resulting in pervasive biases along SDMs of different species, as spatially-structured sampling biases are often shared by all species pertaining to the same group. Implementing robust methods to map species distributions and spatial bias is crucial for natural resource management. In particular, two critical points must be faced explicitly: (i) integrating prior knowledge for improving the prediction of species distributions over wide geographical areas, and (ii) quantifying and visualizing the uncertainty associated with species distribution predictions over large geographical scales. Improved knowledge in areas where the modelled species are predicted to spread, along with illustration of uncertainty of predictions in an easily interpretable map, can lead to more effective management strategies^199^. This would allow timely actions to be initiated, both in case of the protection of natural species and the management of invasive species.

### Reporting summary

Further information on research design is available in the Nature Research Reporting Summary linked to this article.

### Supplementary information

Appendix1_VirtualSDM_VS.R

Reporting Summary

## Data Availability

The virtual data used in this paper is free and open and can be generated by the code provided in section “Code availability”. The full set of empirical data can be downloaded at https://www.gbif.org/.

## Code availability

The code used in this paper is available in Appendix 1 and at the following GitHub repository: https://github.com/ducciorocchini/Virtual_species_SDM/.
